# CD31hiEmcnhi Vessels Support New Trabecular Bone Formation at the Frontier Growth Area in the Bone Defect Repair Process

**DOI:** 10.1038/s41598-017-04150-5

**Published:** 2017-07-10

**Authors:** Jimeng Wang, Yi Gao, Pengzhen Cheng, Donglin Li, Huijie Jiang, Chuanlei Ji, Shuaishuai Zhang, Chao Shen, Junqin Li, Yue Song, Tianqing Cao, Chunmei Wang, Liu Yang, Guoxian Pei

**Affiliations:** Institute of Orthopaedics and Traumatology, Xijing Hospital, Fourth Military Medical University, Xi’an, 710032 People’s Republic of China

## Abstract

CD31hiEmcnhi vessels were a subtype of vessels in the murine skeletal system, with high levels of platelet and endothelial cell adhesion molecule-1 (PECAM-1/CD31) and endomucin (Emcn). They were reported coupling angiogenesis and osteogenesis during bone development. We investigated the distribution of these vessels in rat tibiae and their temporal and spatial distribution during the bone defect repair process to improve our understanding of the importance of these vessels. We confirmed that CD31hiEmcnhi vessels were specially distributed around the trabecular bones near metaphysis and endosteum in rat tibiae. At 3 days post bone injury, CD31hiEmcnhi vessels proliferated and were extensively distributed across the entire repair area. At 7 and 14 days post-injury, these vessels decreased but were specially distributed around the growing trabecular bones near the frontier growth area, suggesting that these vessels support new bone formation. The distribution of CD31hiEmcnhi vessels and the transcriptions of *Hif*-*1α* and *VEGFA*, as well as *BMP2* and *Osterix* decreased at 7 and 14 days post-injury under osteoporotic conditions, in combination with insufficient osteogenesis. Our research is of great significance to help understand the important role of CD31hiEmcnhi vessels in supporting new trabecular bones formation during bone defect repair process.

## Introduction

The repair of injured bone involves a series of complex physiological and pathological processes. Four typical events occur during bone injury repair process: hematoma formation, organization of hematoma, primary trabecular bone formation and bone reshaping and remodeling^1–3^. New blood vessels formation, or angiogenesis, is an important event in the hematoma organization process, and some studies have suggested that angiogenesis occurs prior to the onset of osteogenesis^4, 5^. These new vessels supply the regenerating tissue, which has a high metabolic rate, with nutrients, remove waste products, and provide growth factors, cytokines and chemokines^6^. They also provide access routes for the surrounding and circulating repair cells, such as fibroblasts, stem cells, osteoprogenitors and endothelial cells, which migrate into the injured site^7–9^. Thus, angiogenesis provides the callus with nutrients, repair cells, growth factors, and an appropriate growth microenvironment for bone defect repair^10^.

Previous researchers have suggested a close link between angiogenesis and osteogenesis based on their close spatial and temporal association^5, 9, 11, 12^. Signal transmission or crosstalk between vascular endothelial cells and osteoprogenitor cells has also been demonstrated^9, 13^. This association is called “angiogenic-osteogenic coupling”^9^. In the murine skeletal system, Kusumbe *et al*.^14^ recently reported a special capillary subtype, CD31hiEmcnhi, which exhibited distinct morphological, molecular and functional properties. These special capillaries subtype were primarily distributed within the metaphysis and endosteum area, where cells maintain their growth and proliferation abilities and sustain bone development. Notably, these researchers also found that the number of CD31hiEmcnhi vessels and their surrounding osteoprogenitors were greatly reduced with age, especially in elderly mice. Their further research^15^ revealed that the endothelial Notch signaling, noggin and vascular endothelial growth factor (VEGF) coupled angiogenesis and osteogenesis during bone development. Hui Xie *et al*.^16^ demonstrated that the abundance of CD31hiEmcnhi vessels decreased significantly in ovariectomized (OVX) mice and that the secretion of platelet-derived growth factor-BB (PDGF-BB) by preosteoclasts induced CD31hiEmcnhi vessels growth during bone modeling and remodeling.

The promotion of osteogenesis by CD31hiEmcnhi vessels is important during the process of bone development in mice. However, the distribution of CD31hiEmcnhi vessels in the adult rat skeletal system is not clear, and the distribution and role of CD31hiEmcnhi vessels during the bone defect repair process under normal and osteoporotic conditions remain unknown. Our research focused on the close spatial and temporal associations between new vessels and migrating osteoprogenitors during the repair process, and investigated the differences in the proliferation and distribution of CD31hiEmcnhi vessels during the bone defect repair process under normal and osteoporotic conditions. We confirmed that there were CD31hiEmcnhi vessels distributing around the trabecular bones near the metaphysis and endosteum in rat tibiae. The proliferation and distribution of CD31hiEmcnhi vessels exhibited temporal-spatial specificity during the process of bone defect repair, suggesting that these vessels support new bone formation. We also compared the differences in the transcriptional levels of angiogenesis- and osteogenesis-related factors between normal and osteoporotic rats during the process of bone defect repair. We found that the defective CD31hiEmcnhi vessels formation and low transcriptional levels of *hypoxia*-*inducible factor 1α* (*Hif*-*1α*), *VEGFA*, *bone morphogenetic protein*-*2* (*BMP2*) and *Osterix* after bone defect were closely related to the delayed osteogenesis under osteoporotic conditions.

## Results

### The repair process of the tibia drilling bone defect model involved typically intramembranous ossification

**Figure 1:**
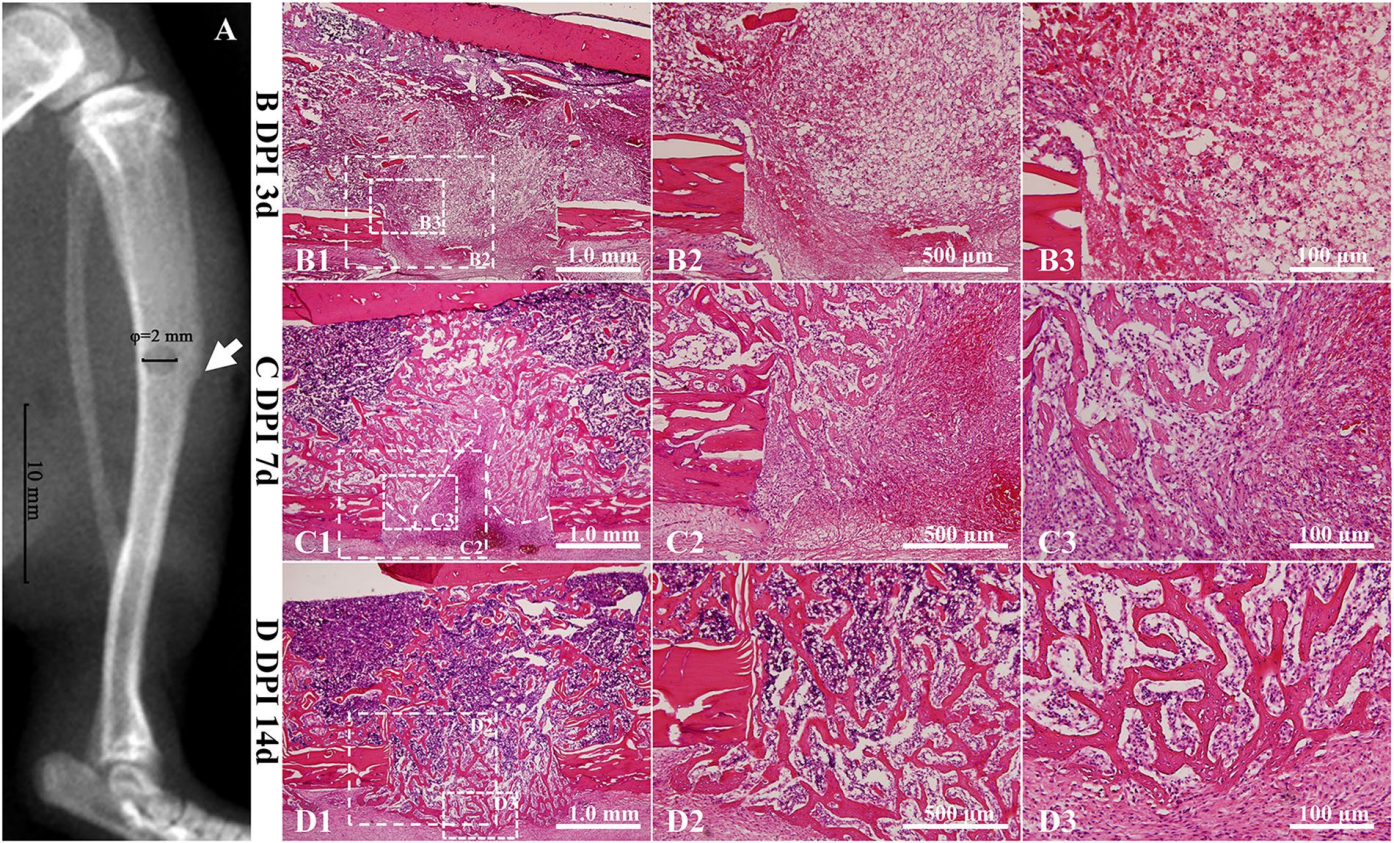
The drilling site of the tibia bone defect and the repair process at different time points. (**A**) X-ray of the tibia bone defect showing the drilling site. The white arrow was pointed to the tibial crest. The diameter of the hole is 2 mm. (**B**–**D**) H/E staining of the injured region at 3, 7 and 14 days post-injury. DPI: days post-injury. (**B**). The hematoma has been well organized at 3 days post-injury, with plenty of vascular tissues invading into and a large number of cells migrating into the inner. (**C**) As the dotted line showed, the frontier growth area could be identified at 7 days post-injury. The thickness of trabecular bones near the frontier growth area was relatively small compared with those away from the frontier growth area. Osteoblasts could be found at the surface of trabecular bones. Vascular structures existed both at the surface and periphery of trabecular bones. A large number of cells accumulated at the forward of frontier growth area and new trabecular bones was forming. (**D**) Newly formed trabecular bones have bridged the bilateral cortical bones at 14 days post-injury. At this time point, the repair period was in the process of bone remodeling stage. Bone marrow cavity has been gradually recanalized.

A series of physiological reactions were triggered immediately after bone injury to limit damage and restore the bone. Hematoxylin and eosin (H/E) staining revealed a highly organized granulation tissue that contained abundant cells at 3 days post-injury (Fig. 1B1). Diverse cells, such as fibroblasts, inflammatory cells, endothelial cells and osteoprogenitors, were distributed throughout the injured area (Fig. 1B2). Proliferating and migrating cells could be identified at the edge of the bone defect, which were more likely derived from the nearby endosteum (Fig. 1B3). Plenty of trabecular bones had been formed inside the injured region at 7 days post-injury. The trabecular bones near the frontier growth area exhibited smaller volume and thickness, while the trabecular bones away from the frontier growth area exhibited larger volume and thickness (Fig. 1C1). The overall shape of new trabecular bones was consistent with the migration of proliferating endosteal cells. Blue-stained osteoblasts adhered on the surface of trabecular bones. Osteoblasts secreted osteoid and embedded themselves in the bone lacunae. Vascular structures were distributed both at the surface and among the trabecular bones. Osteoid structures could be observed at the front of the frontier growth area, confirming the formation of new bones (Fig. 1C3). More trabecular bones were formed and connected the bilateral cortical bones at 14 days post-injury. The volume and thickness of the trabecular bones at the center region became small, indicating that the bones were undergoing resorption and that the repair process was in the bone remodeling and reshaping stage. The marrow cavity began to link together at this time point (Fig. 1D1). H/E staining revealed no chondrocyte formation inside the injured hole during the repair process.

### Osterix + Emcn+ cells mediated new bone formation

**Figure 2:**
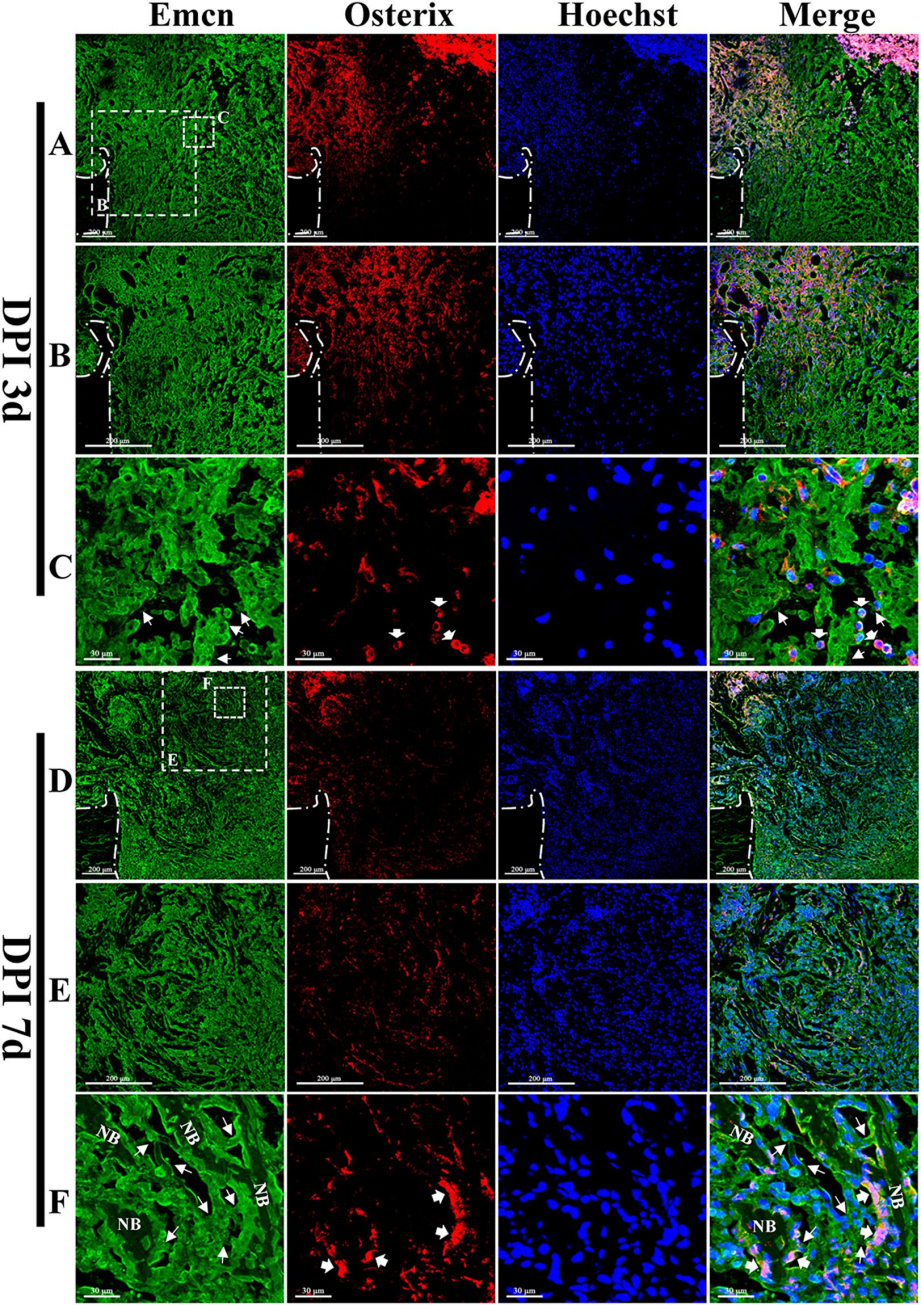
The invasion of Emcn+ capillaries and the proliferation and migration of osterix+ cells during the repair process. (**A**–**C**) The distribution of Emcn+ vessels and osterix+ cells at 3 days post-injury. (**A**) Emcn+ vessels were extensively distributed in the injured area, exhibiting a denser distribution in the lateral area and looser distribution in the inner area. (**B**) Proliferating and migrating osterix+ cells were primarily distributed at the lateral side of the repairing region, and displayed a migrating tendency to the inner. (**C**) The sprouting of Emcn+ capillaries could be observed inside the repair area (white thin-tailed arrow). Some osterix+ cells were tightly connected with Emcn+ capillaries (white thick-tailed arrow). (**D**–**F**) The distribution of Emcn+ capillaries and osterix+ cells at 7 days post-injury. (**D**) Emcn+ capillaries were distributed in a more orderly manner, and were observed both at the surface and the periphery of newly formed trabecular bones. Near the frontier growth area distributed a large number of osterix+ cells. (**E**) Most osterix+ cells were distributed on the surface of newly formed trabecular bones. (**F**) Branches of Emcn+ vessels among the new trabecular bones could be observed (white thin-tailed arrow). Osterix+ Emcn+ cells were identified and were distributed at the surface of new trabecular bones (white thick-tailed arrow).

Emcn antibody was used to label capillaries, and osterix antibody was used to label osteoprogenitors and osteoblasts. Emcn/osterix double fluorescent labeling revealed how new vessels grew and osteoprogenitors migrated into the center of the injured zone and showed their distribution and location relationship. At 3 days post-injury, Emcn+ capillaries were widely distributed in the injured zone (Fig. 2A). Capillaries at the center of the injured zone were fewer in number and were more porous than those at the lateral (Fig. 2B). Capillary sprouting could be observed near the center of the injured zone, indicating angiogenesis was happening (Fig. 2C). Osterix+ cells were primarily distributed in the endosteal region near the damaged cortical bones, while only a few scattered cells were distributed at the center of the injured zone. The osterix+ cells were most likely derived from endosteum and bone marrow (Fig. 2B). Emcn+ capillaries were distributed extensively in the injured zone at 7 days post-injury and were more orderly than those observed at 3 days post-injury (Fig. 2D,E). Emcn+ capillaries were distributed both at the surface and the periphery of trabecular bones (Fig. 2E). The capillaries at the periphery of trabecular bones branched at the end and connected with the capillaries at the surface of trabecular bones (Fig. 2F). As expected, a large number of osterix+ cells were present around the newly formed trabecular bones, indicating that the trabecular bones were formed by osterix+ cells secreting osteoid. Some osterix+ cells were present attaching on the capillaries (Fig. 2E,F). Notably, some osterix+ Emcn+ cells were distributed at the edge of newly formed trabecular bones and likely mediated new bone formation (Fig. 2C,F). Emcn protein was identified as an endothelial sialomucin expressed on the surface of endothelial cells. The co-localization of osterix and Emcn may indicate the endothelial characteristics of osteoblasts, or as microcapillary pericytes, which were directly connected with their supporting capillaries. The exchange of nutrients, oxygen, metabolic products and endocrine factors occurred between capillaries and osterix+ Emcn+ cells.

### CD31hiEmcnhi vessels were distributed especially in areas with high levels of metabolic and osteogenic activity

**Figure 3:**
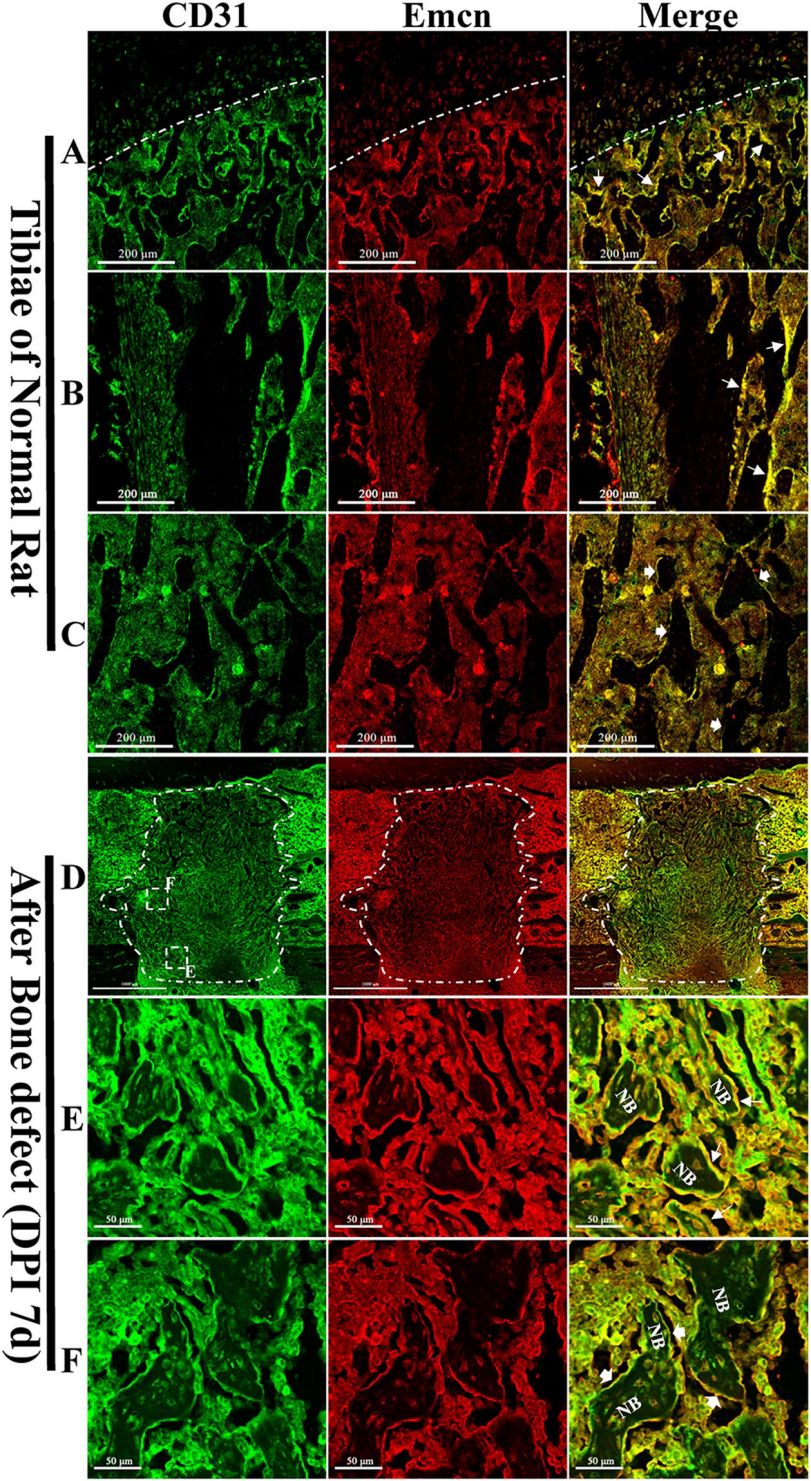
The distribution of CD31hiEmcnhi vessels had spatial specificity both in normal rat tibiae and the injured tibiae during repair process. (**A–C**) The distribution of CD31hiEmcnhi vessels in different parts of normal rat tibiae. (**A**) Metaphysis. (**B**) Diaphysis. (**C**) Bone marrow cavity. Plenty of CD31hiEmcnhi vessels were distributed around the growing trabecular bones near metaphysis and endosteum areas (white thin-tailed arrow). While at the surface of trabecular bones inside the bone cavity distributed only a few CD31hiEmcnhi vessels (white thick-tailed arrow). (**D**–**F**) CD31hiEmcnhi vessels distributed differently at different portion of the repair area at 7 days post-injury. (**D**) The overall appearance of CD31/Emcn double-immunofluorescence staining at 7 days post-injury showed the distribution of CD31hiEmcnhi vessels. (**E**) High levels of CD31hiEmcnhi vessels were distributed at the surface of the growing trabecular bones near the frontier growth area. (**F**) While at the surface of trabecular bones away from the frontier growth area exhibited a low level distribution of CD31hiEmcnhi vessels.

The distribution of CD31hiEmcnhi vessels in rat bone exhibited spatial specificity. These vessels were highly distributed in areas with high levels of metabolic and osteogenic activity. High levels of CD31hiEmcnhi vessels were distributed at the surface of the growing trabecular bones near metaphysis and endosteum in the rat tibiae (Fig. 3A,B), while only a few CD31hiEmcnhi vessels were distributed at the surface of the trabecular bones inside the bone marrow cavity (Fig. 3C). These appearances were similar with what described in the previous report. Capillaries were observed both at the surface and the periphery of trabecular bones at 7 days post-injury. Notably, the trabecular bones near the frontier growth area were surrounded by high levels of CD31hiEmcnhi vessels (Fig. 3D,E), while relatively few CD31hiEmcnhi vessels were observed at the surface of the trabecular bones away from the frontier growth area (Fig. 3D,F). It was the fact that bones near the frontier growth area were undergoing bone formation, which had a high metabolic and osteogenic activity. While bones away from the frontier growth area were more likely undergoing bone resorption and remodeling (Fig. 3D). The high distribution of CD31hiEmcnhi vessels at the surface of the growing new trabecular bones suggested that CD31hiEmcnhi vessels could support new bone formation.

### CD31hiEmcnhi vessels proliferated in the bone defect repair process supporting new trabecular bones formation

**Figure 4:**
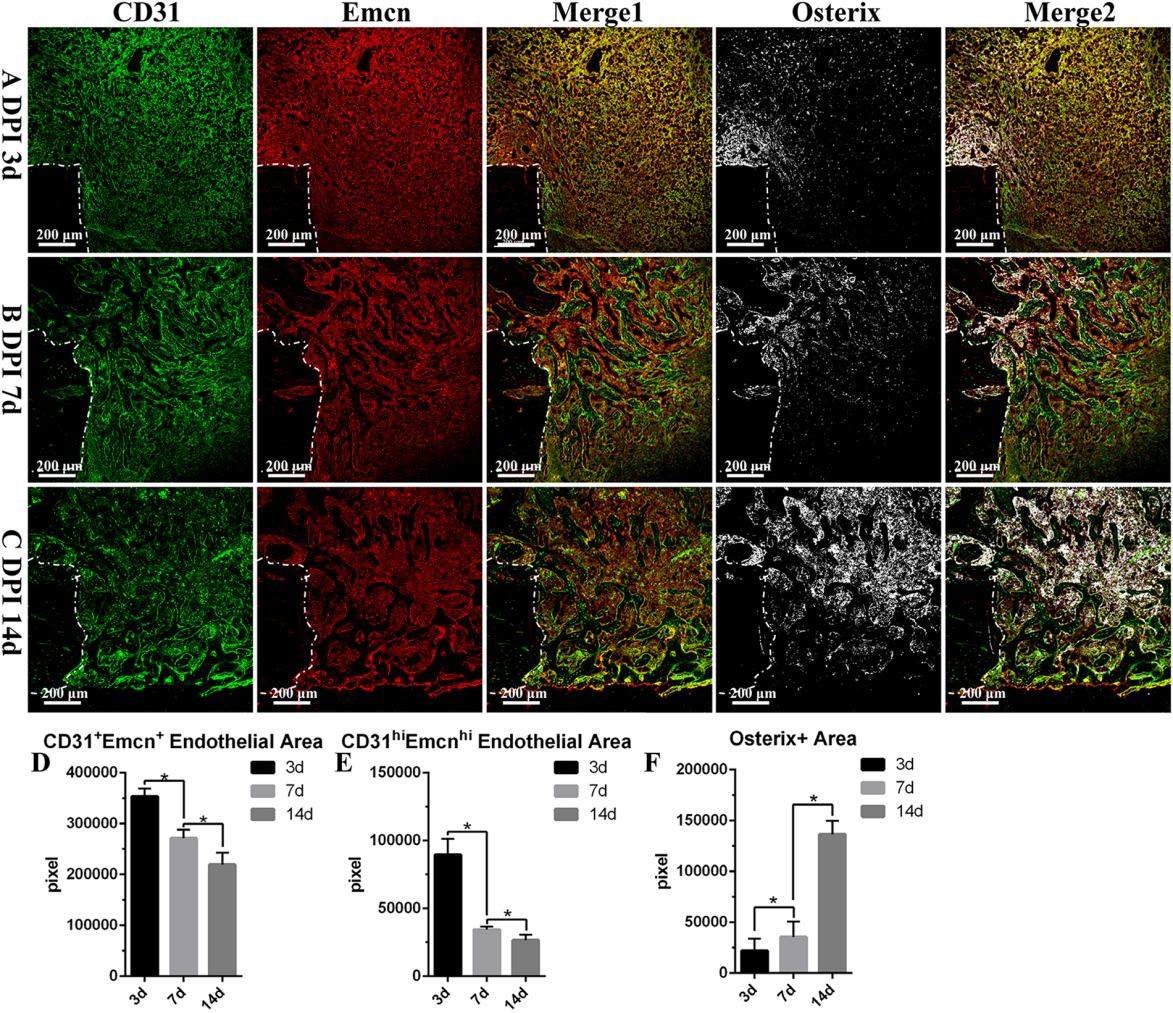
The proliferation and distribution of CD31hiEmcnhi vessels and osterix+ cells at different time points during the bone defect repair process in normal rats. (**A**) High levels of CD31hiEmcnhi vessels were distributed across the whole repair area at 3 days post-injury. A large number of osterix+ cells were concentrated near endosteum at the damaged edge. (**B**) Vessels could be identified both at the surface and the periphery of trabecular bones at 7 days post-injury. Relatively high levels of CD31hiEmcnhi vessels were distributed at the surface of the growing trabecular bones near the frontier growth area. Most osterix+ cells were distributed at the surface of trabecular bones and some were attached on vessels. (**C**) High levels of CD31hiEmcnhi vessels were distributed at the surface of the growing trabecular bones near the lateral margin of cortical bone at 14 days post-injury. While at the surface of trabecular bones inside the cavity distributed very low level of CD31hiEmcnhi vessels. (**D–F**) Statistical comparisons of CD31 + Emcn+ endothelial area, CD31hiEmcnhi endothelial area and osterix+ area at 3, 7 and 14 days post-injury. *P < 0.05, n = 4.

The distribution of CD31hiEmcnhi vessels exhibited temporal-spatial specificity during the bone defect repair process. At 3 days post-injury, the regenerating vessels have invaded into the injured region. The proportion of CD31hiEmcnhi vessels was very high (Fig. 4A). Well-organized tissues have replaced the hematoma at this time point. Abundant new vessels and proliferating and migrating osterix+ osteoprogenitors or osteoblasts were distributed across the repair region (Fig. 4A). At 7 days post-injury, the number of CD31hiEmcnhi vessels decreased, but were specially distributed at the surface of the growing trabecular bones near the frontier growth area (Fig. 4B). Relatively few CD31hiEmcnhi vessels could be observed at the surface of the trabecular bones away from the frontier growth area. Osterix+ cells were not only distributed at the surface of trabecular bones, but were also attached on the vascular structure. At 14 days post-injury, the number of CD31hiEmcnhi vessels further decreased. Only at the surface of the trabecular bones near the lateral margin of cortical bone distributed high levels of CD31hiEmcnhi vessels. While at the surface of the trabecular bones inside the bone marrow cavity distributed few CD31hiEmcnhi vessels (Fig. 4C). Although the number of CD31hiEmcnhi vessels gradually decreased during the repair process, the vessels were specially distributed at the surface of the growing trabecular bones. These results indicated that CD31hiEmcnhi vessels support new trabecular bones formation during the bone defect repair process. Proliferating and migrating osterix+ osteoprogenitors or osteoblasts were primarily derived from the endosteum near the defect edge at 3 days post-injury (Fig. 4A). There were plenty of osterix+ cells distributed inside the vascular structure of bone marrow cavity, indicating the high proliferation of osterix+ cells at 14 days post-injury (Fig. 4C). The area of osterix+ cells significantly increased during the repair process (Fig. 4F).

### The decreased bone mass in the process of bone defect repair under osteoporotic conditions was related with the reduction of CD31hiEmcnhi vessels and low transcriptional levels of Hif-1α, VEGFA, BMP2 and osterix

**Figure 5:**
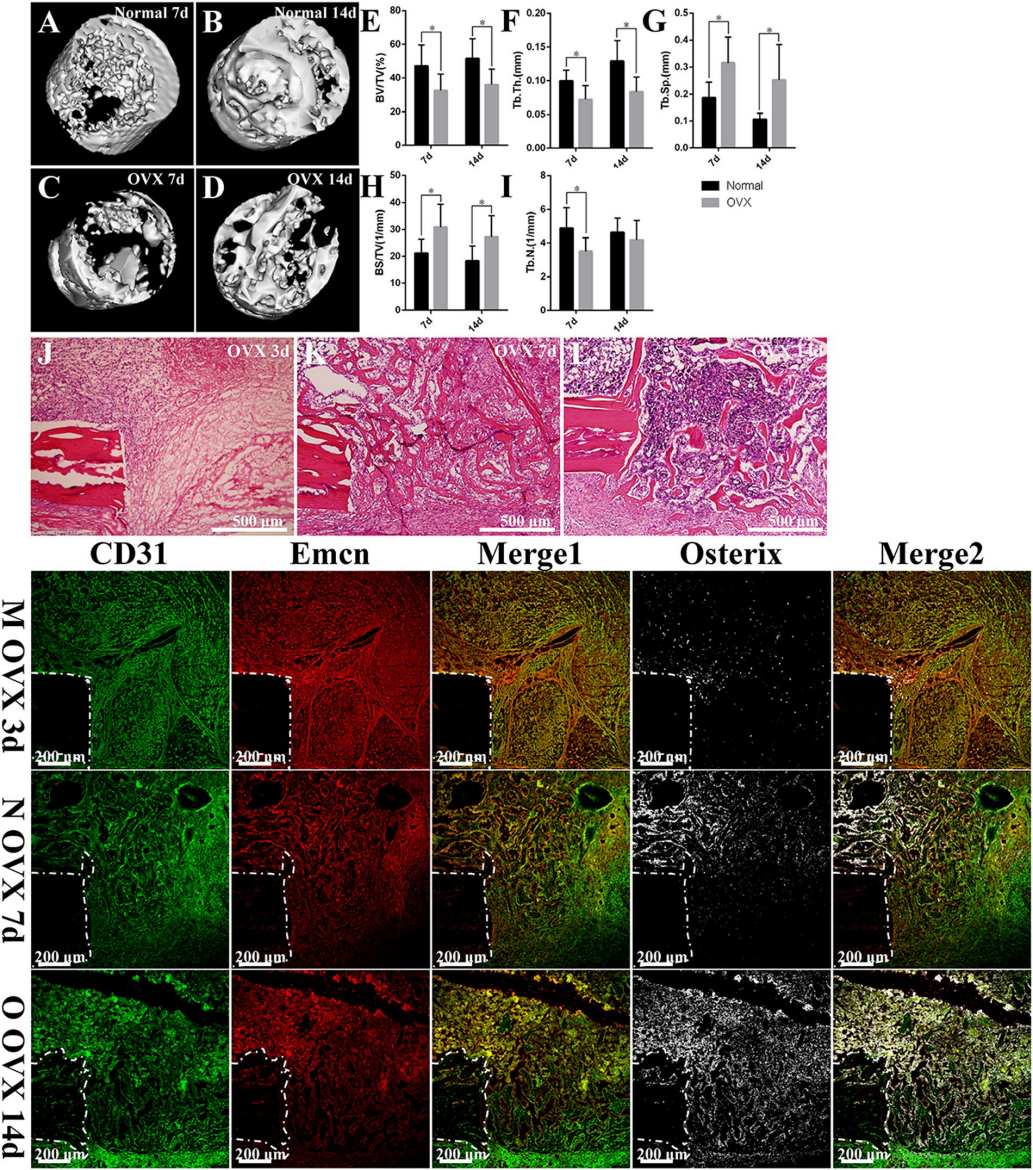
The repair process of the tibia drilling bone defect model in OVX rats. (**A–D**) Three-dimensional reconstruction after micro-CT scanning showed that new bones formations during the repair process at 7 and 14 days post-injury in OVX rats were lower than those in normal rats. (**E–I**) Statistical comparisons of BV/TV, Tb.Th., Tb.Sp., BS/TV and Tb.N. between normal rats and OVX rats at 7 and 14 days post-injury. *P < 0.05, n = 4. (**J–L**) H/E staining at different time points in the repair process of tibia drilling bone defect in OVX rats. The degree of hematoma organization at 3 days post-injury in OVX rats was obviously inadequate compared with that in normal rats (Fig. 1). The thickness of new trabecular bones was reduced at 7 days post-injury in OVX rats. The number and thickness of trabecular bones reduced, but the space increased at 14 days post-injury in OVX rats. (**M–O**) The proliferation and distribution of CD31hiEmcnhi vessels and osterix+ cells at different time points during the bone defect repair process in OVX rats. Compared with the repair process in normal rats, the area of CD31hiEmcnhi vessels was decreased at every time points in OVX rats. The high distribution of CD31hiEmcnhi vessels at the surface of trabecular bones near the frontier growth area at 7 days post-injury was not obvious. There was no high distribution of CD31hiEmcnhi vessels at the surface of the trabecular bones near the lateral margin of cortical bone at 14 days post-injury. The areas of osterix-staining positive at the repair region were decreased at 3, 7 and 14 days post-injury in OVX rats compared with those in normal rats.

**Figure 6:**
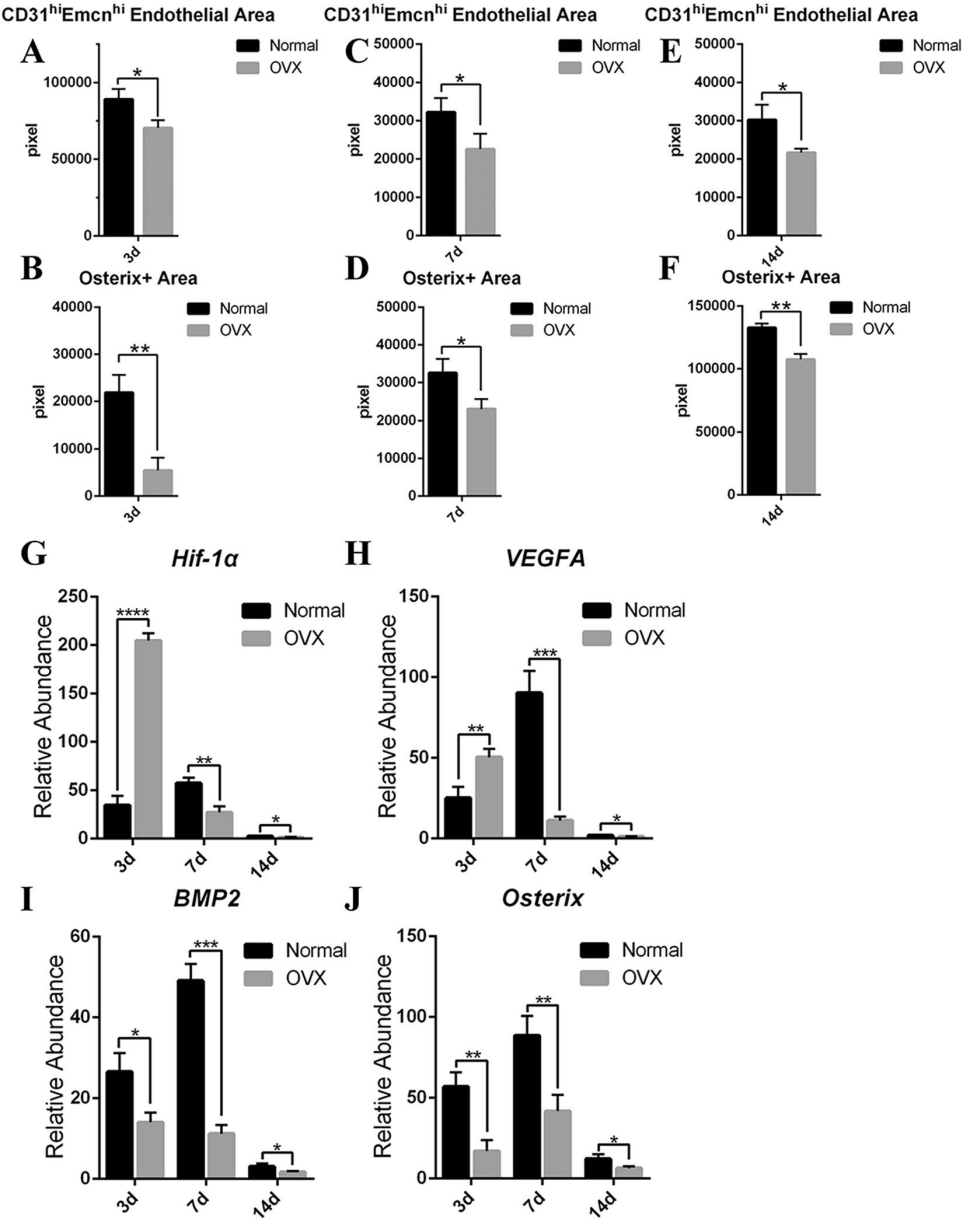
(**A–F**) Statistical comparisons of CD31hiEmcnhi vessels and the area of osterix-staining positive between normal and OVX rats during the repair process. Under osteoporotic conditions, the amounts of CD31hiEmcnhi vessels and osterix+ cells were significantly reduced at 3, 7 and 14 days post-injury. (**G–J**) Statistical comparisons of the transcriptional levels of *Hif*-*1α*, *VEGFA*, *BMP2* and *Osterix*. The transcriptions of *Hif*-*1α* and *VEGFA* at 3 days post-injury in OVX rats were significantly higher than those in normal rats. *P < 0.05; **P < 0.01; ***P < 0.001, n = 4.

Micro-computed tomography (CT) analyses revealed that the bone volume fraction (bone volume/total volume, BV/TV), trabecular thickness (Tb.Th.) and trabecular number (Tb.N.) decreased significantly at 7 and 14 days post-injury in OVX rats, whereas the bone surface-to-volume ratio (bone surface/total volume, BS/TV) and trabecular spacing (Tb.Sp.) significantly increased (Fig. 5A–I). These changes demonstrated that the osteogenesis after bone defect in OVX rats was significantly insufficient relative to that in normal rats. H/E staining revealed that the degree of hematoma organization was significantly reduced at 3 days post-injury under osteoporotic conditions. A large number of proliferating cells accumulated at the edge of the defect. While only a small number of cells migrated into the inner portion of the repairing tissue. These results may be attributed to the lack of neovascularization (Fig. 5J). The number and thickness of newly formed trabecular bones decreased in OVX rats at 7 days post-injury, consistent with the results of micro-CT scanning. The trabecular structure was disordered compared with that in normal rats (Fig. 5K). Similarly, the number and thickness of trabecular bones at 14 days post-injury in OVX rats reduced significantly, while the space increased significantly. The entire trabecular structure was loose and porous in OVX rats compared with that in normal rats (Fig. 5L). Immunofluorescent staining revealed that the area of CD31hiEmcnhi vessels was decreased at each period of the repair process under osteoporotic conditions (Fig. 6A,C,E). The high distribution of CD31hiEmcnhi vessels at the surface of the trabecular bones near the frontier growth area was not obvious under osteoporotic conditions (Fig. 5N,O). In OVX rats, the number of osterix+ osteoprogenitors or osteoblasts decreased significantly at 3 days post-injury, and this decrease was accompanied by insufficiently invading of new blood vessels (Fig. 6B). Additionally, only a small number of osterix+ cells migrated into the center of the repairing tissue (Fig. 5M). The volume of vascular tissues at the surface and the periphery of trabecular bones was decreased in OVX rats at 7 days post-injury (Fig. 5N). In OVX rats, the high distribution of CD31hiEmcnhi vessels at the surface of the trabecular bones near the lateral margin of cortical bone was not obvious at 14 days post-injury (Fig. 5O). Obvious nonspecific staining of bone marrow was excluded when calculating the fluorescence intensity.

We detected the differences in the transcriptional levels of the angiogenesis-related factors *Hif*-*1α* and *VEGFA*, as well as the osteogenesis-related factors *BMP2* and *Osterix* during the repair process, to interpret the low level of angiogenesis and osteogenesis in OVX rats at the mRNA transcriptional level. Surprisingly, we found that, compared with those in normal rats, the transcriptions of *Hif*-*1α* and *VEGFA* in OVX rats were not decreased at 3 days post-injury, but were increased significantly (P < 0.05) (Fig. 6G,H). However, the transcriptional levels of *Hif*-*1α* and *VEGFA* at 7 and 14 days post-injury in OVX rats were decreased significantly compared with those in normal rats (P < 0.05) (Fig. 6G,H). The transcriptional levels of the osteogenesis-related factors *BMP2* and *Osterix* were consistent at each time point after bone defect. Under osteoporotic conditions, the transcriptional levels of *BMP2* and *Osterix* were significantly lower than those under normal conditions (Fig. 6I,J). The transcriptional levels of *Hif*-*1α*, *VEGFA*, *BMP2* and *Osterix* in normal rats reached peak values at 7 days post-injury (Fig. 6G–J). These results suggested that the significant decrease in the transcriptional levels of angiogenesis- and osteogenesis- related factors at 7 and 14 days post injury possibly led to low levels of angiogenesis and osteogenesis in OVX rats.

## Discussion

Our study focused on the dynamic distribution of CD31hiEmcnhi vessels during the bone defect repair process in normal adult rats and osteoporotic rats. Immunohistochemical staining at multiple time points revealed the proliferation and distribution of CD31hiEmcnhi vessels after bone injury. Combined with the research of Kusumbe *et al*., we showed that CD31hiEmcnhi vessels support new trabecular bones formation during the bone defect repair process in adult rats. We firstly detected the distribution of CD31hiEmcnhi vessels in normal rat tibiae and found that these vessels were highly located at the surface of trabecular bones near metaphysis and endosteum, where stem cells and progenitor cells maintain their self-renewal capacity and allow lifelong osteogenesis. This finding is similar to the previous results obtained in mice. Next, we found that the distribution of CD31hiEmcnhi vessels displayed temporal-spatial specificity during the repair process. At 3 days post-injury, when the hematoma was organized with abundant new vessels, high levels of CD31hiEmcnhi vessels were widely distributed across the injured region. Subsequently, CD31hiEmcnhi vessels decreased at 7 and 14 days post-injury but were specially distributed at the surface of the growing trabecular bones near the frontier growth area. By contrast, at the surface of the trabecular bones away from the frontier growth area distributed low levels of CD31hiEmcnhi vessels. These observations indicated that CD31hiEmcnhi vessels could support new trabecular bone formation during the repair process. As a contrast, under osteoporotic conditions, the distribution of CD31hiEmcnhi vessels was reduced at every period of the repair process. There were no obvious high levels of CD31hiEmcnhi vessels distributed at the surface of the new trabecular bones near the frontier growth area at 7 and 14 days post-injury in OVX rats. Meanwhile, the bone mass and bone quality repaired in OVX rats were decreased. Kusumbe *et al*.^14^ once detected the transcriptional levels of exocrine factors related to the proliferation and survival of osteoprogenitors in mice and found that the transcriptional levels of *Pdgfa*, *Pdgfb*, *Tgfb1*, *Tgfb3 and Fgf1* were significantly higher in CD31hiEmcnhi endothelial cells. Selectively blocking the endothelial Notch signaling^15^ reduced the distribution of CD31hiEmcnhi vessels, shortened long bones of mice and induced bone loss. These findings further indicated the hypothesis that CD31hiEmcnhi vessels could support new trabecular bone formation in the repair process. Although more underlying mechanisms remained to be explored, the high proliferation and distribution of CD31hiEmcnhi vessels during the bone defect repair process may represent a highly organized and metabolized repair tissue that could influence the bone growth potential.

Our research confirmed that new vessels invasion occurred predominantly before the onset of osteogenesis after bone defect. Vessels are damaged after injury, leading to hematoma formation^17^ and innate immune response activation^4, 18^. Hypoxia, fibrin matrix^19^, inflammatory cells and cytokines are strong inducers of endothelial cells^20–23^. New vessels begin sprouting and invading into the hematoma. We found that at 3 days post-injury Emcn+ capillaries were distributed extensively throughout the repair region, while only a small number of osterix+ cells migrated into. A large number of proliferating osterix+ cells were found accumulating at the defect edge. Notably, we found that the invading osterix+ cells co-expressed the endothelial sialomucin protein-Emcn and were located at the edge of capillaries. This co-expression indicates a close connection between osteoblasts and capillaries. Similarly, Maes *et al*.^24^ reported that osterix+ osteoblast precursors moved into developing bones along with the invading vessels in pericyte-like fashion during endochondral bone development and endochondral healing of bone fractures, and that the pericyte-like osterix+ cells gave rise to the full differentiation spectrum of osteoblast lineage cells inside the growing bone, including the cuboidal osteoblasts covering the trabecular bone surfaces. Histological and transmission electron microscope (TEM) analysis revealed that the osterix+ cells were juxtaposed to endothelial cells and their processes closely enfolded the endothelial lining of the vessel, similar to pericytes. These findings are consistent with the characterization of osterix+ cells as a type of microvascular pericytes closely affiliated with capillary endothelial cells^24, 25^. However, whether osterix+ cells migrate into the specific locations, via extravascular migratory metastasis attaching on vessels or trans-endothelial migration similar to hematopoietic cells is not known. At 7 days post-injury, mature vessels inside the injured area had formed. The capillary tubes among trabecular bones are branched at the end and connected to osterix+ bone-forming cells^8^. These structures ensure that the osteoblasts could receive a continuous supply of nutrients and oxygen to perform their functions. Previous studies have shown that in addition to the cambial layer of the periosteum and mesenchymal stem cells in bone marrow, endosteal cells and circulating progenitors are important sources of osterix+ cells^26–28^. In our study, we did not trace the sources of the osterix+ cells. However, the location of the osterix+ area suggests that these cells were primarily derived from proliferating and migrating endosteal cells near the bone defect edge, of course bone mesenchymal stem cells cannot be excluded.

Osteoporosis represents an imbalance between osteoblastic bone formation and osteoclastic bone resorption resulting from long-term chronic inflammation and excessive osteoclast activity^29^. The ultimate results are bone volume loss, bone strength decrease and a high risk of bone fracture^30, 31^. *Hif*-*1α* is the most important regulator of oxygen homeostasis in mammals and controls physiological and pathological neo-angiogenesis^32^. Under acute hypoxia conditions, the transcription of *Hif*-*1α* and the stability of Hif-1α protein significantly increase. Binding of Hif-1α to the hypoxia response element (HRE) activates the transcriptions of target genes such as *VEGF* ^33^. VEGFA is the most important vascular endothelial growth factor, and has the strongest effect on inducing angiogenesis by binding to VEGF receptors (VEGFRs) on the surface of endothelial cells^34^. Researchers previously observed reduced bone vasculature and decreased Hif-1α and VEGF expression in the bones of OVX mice^35–37^. Our study detected that at 3 days post-injury the transcriptional levels of *Hif*-*1α* and *VEGFA* in OVX rats were significantly higher than that in normal rats. However, at 7 and 14 days post-injury the transcriptional levels of *Hif*-*1α* and *VEGFA* in OVX rats reduced and were significantly lower than that in normal rats. Our latter results were similar with the report of Li *et al*.^36^ in which they found decreased callus formation, reduced neovascularization and low levels of *Hif*-*1α* transcription in OVX rats at 14 and 28 days post-femoral fracture compared with normal rats. We attribute the high transcriptional levels of the angiogenesis-related factors *Hif*-*1α* and *VEGFA* at 3 days post-injury under osteoporotic conditions to the low oxygen tension of the repairing callus due to poor neovascularization in OVX rats. By contrast, in normal rats, the hematoma was well organized, and the callus was relatively rich in new vessels and oxygen. The reasons why high transcriptions of *Hif*-*1α* and *VEGFA* did not result in good angiogenesis in OVX rats require further investigation. BMPs play an important role in stimulating the differentiation of mesenchymal stem cells into osteoblasts, and BMP2 is one of the most important BMPs^38^. *Osterix* is a specific transcription factor of osteoprogenitors and osteoblasts, whose transcription is positively regulated by BMP2^39^. The decreases in the transcriptional levels of *BMP2* and *Osterix* were consistent with the low bone mass and reduced number of osterix+ cells at 3, 7 and 14 days post-injury in OVX rats.

In summary, our study is of great significance for understanding the importance of the proliferation, distribution and function of CD31hiEmcnhi vessels during the bone defect repair process. However, further investigation is required to obtain deeper knowledge of the mechanisms underlying the appearance of these vessels. Increasing the number and distribution of CD31hiEmcnhi vessels during the bone defect repair process using drug treatment or physical therapy could be important for the promotion of bone defect repair under osteoporosis and constitutes one direction of our further research.

## Methods

### Animal models

A tibia drilling bone defect model was used in this experiment. Surgeries were performed on 5-month-old adult female Sprague-Dawley (SD) rats weighing 280 to 320 g. The rats were anesthetized with intraperitoneal sodium pentobarbital (45 mg/kg), shaved, and placed in a supine position on the surgery table. The lower right limb was disinfected, and the skin above the tibia was cut along the bone edge. A drill bit (φ = 2 mm) was used to drill a hole in the ventral side of the tibia at the crest level. The hole did not penetrate the other side of the bone. The incised skin was carefully sutured, and the rat was placed on a heating pad until awake. Ampicillin (100 mg/kg) was injected intraperitoneally once daily for 3 consecutive days. The rats were randomly divided into 3 groups of 8 rats each based on the time of euthanasia: 3 days, 7 days and 14 days post-injury. Ovariectomies were performed on 2-month-old female SD rats according to the methods of Wronski^40^. Tibia drilling bone defect surgeries were performed on OVX rats 3 months later. The OVX rats were grouped as described above. The laboratory animal center of the Fourth Military Medical University provided all rats. The rats were maintained in specific pathogen-free (SPF)-class housing in the laboratory. The Ethical Committee of the Fourth Military Medical University approved this study. All procedures on rats were performed in the authorized animal care facility and were approved by the “Committee for the Care and Use of Laboratory Animals” of the Fourth Military Medical University. All methods were performed in accordance with the relevant guidelines and regulations of the People’s Republic of China.

### Tibiae collection and decalcification

Four rats from each group at each time point were deeply anesthetized with intraperitoneal sodium pentobarbital (100 mg/kg). The chest was opened to expose the heart, and 500 ml of ice-cold 0.9% physiological saline was infused through the left ventricle and ascending aorta, followed by 500 ml of ice-cold 4% paraformaldehyde (PFA) in 0.1 M phosphate-buffered saline (PBS). The soft tissue of the right tibia was removed, and the injured portion of the tibia was collected. Decalcification was performed using 20% ethylenediaminetetraacetic acid (EDTA) at room temperature under constant shaking on a swing bed for 2 months.

### Micro-CT analysis

The fixed tibiae were collected and scanned using a micro-CT system (GE eXplore Locus SP) with a voltage of 80 kV, intensity of 80 mA, integration time of 3000 ms, and layer thickness of 14 μm. Data were analyzed using IPL V5.15 software at the Department of Orthopaedics, Xijing Hospital, China.

### Histochemical staining studies

Decalcified bones were immersed in a solution of 20% sucrose and 2% polyvinylpyrrolidone (PVP) for 72 h. Subsequently, the bones were embedded and frozen in optimal cutting temperature (OCT) compound, and 10-μm sections were longitudinally cut using a Leica CM1850 cryostat. The sections were collected on Superfrost+ microscope slides, and H/E staining was performed using the procedures described in the staining kit. Air-dried sections for immunofluorescent staining were washed 3 times with 0.1 M PBS for 5 min to moisten the slices and wash away any residual OTC. The sections were incubated with 0.01% Triton X-100 for 10 min and washed 3 times with 0.1 M PBS for 5 min. Then, the sections were blocked with 10% donkey serum at 37 °C in an incubator for 60 min. The serum was removed, and the sections were incubated with primary antibodies diluted in 5% donkey serum overnight at 4 °C. The following primary antibodies were used: endomucin (sc-19901, Santa Cruz, diluted 1:100), CD31 (ab64543, Abcam, diluted 1:200) and osterix (ab-22552, Abcam, diluted 1:200). The sections were washed 3 times for 5 min and stained with a secondary anti-goat (1:400, ab150133/ab150136; Abcam), anti-mouse (1:200, 34106ES60; Yesen), or anti-rabbit (1:400, ab150075/ab150076; Abcam) antibody for 1 h at 37 °C. Sections for nuclear staining were incubated with 10 mg/ml Hoechst (33342) for 5 min, washed 6 times with 0.1 M PBS for 5 min, mounted in anti-fade mounting medium (P0126, Beyotime) and coverslipped. A laser scanning confocal fluorescence microscope (FluoView™ FV1000) was used for observation and image capture. The immunofluorescence intensity was quantified using ImageJ software according to a previously described protocol^14^.

### qRT-PCR

Four rats in each group were euthanized. The fresh injured tibias were excised, and the newly formed tissues inside the drilling hole were carefully and quickly collected. All procedures were performed at 4 °C. The total cellular RNA of the tissues was extracted using Trizol Reagent (Invitrogen) according to the manufacturer’s instructions. RNA samples from injured tibial tissues collected from normal and osteoporotic rats at different time points (3, 7 and 14 days) were examined by measuring the ratio of the optical density at 260 nm and 280 nm (OD260/280). Samples with a ratio of 2.0 were used for reverse transcription. cDNA was synthesized using 1 μg of RNA and a Revert Aid First Strand cDNA Synthesis Kit (TaKaRa, Dalian, China). qRT-PCR analyses were performed in triplicate using an SYBR Premix Ex Taq^TM^ kit (TaKaRa, Dalian, China) and detected with Bio-Rad X97 System software. Melting curve analysis was performed for each qRT-PCR assay to minimize nonspecific reactions. Data were normalized to the transcriptional level of *glyceraldehyde 3*-*phosphate dehydrogenase* (*GAPDH*). The primer sequences for the detected genes are listed in Table 1.

**Table 1:** Primer sequences for the detected genes.

Gene	Forward	Reverse
*Hif*-*1α*	5′-ACAAGAAACCGCCTATGACG-3′	5′-TAAATTGAACGGCCCAAAAG-3′
*VEGFA*	5′-GCTCTCTTGGGTGCACTGGA-3′	5′-CACCGCCTTGGCTTGTCACA-3′
*BMP2*	5′-CAGAGCTCCAGATTTTTCGG-3′	5′-CTGGACTTAAGACGCTTCCG-3′
*Osterix*	5′-TGACTGCCTGCCTAGTGTCTACA-3′	5′-TGGATGCCCGCCTTGT-3′
*GAPDH*	5′-CAGCAAGGATACTGAGAGCAAGAG-3′	5′-GGATGGAATTGTGAGGGAGATG-3′

### Statistical analysis

All data were presented as means ± standard deviations. Two-tailed Student’s t test was used to calculate the significance of differences between groups. P < 0.05 was considered statistically significant. GraphPad Prism software (Version 6.01) was used for all statistical analyses.
